# Response of Elite Onion Genotypes to Drought Stress: Morphophysiological and Agronomic Parameters and Stress Indexes

**DOI:** 10.1002/pei3.70099

**Published:** 2025-11-28

**Authors:** Oladé Charles Sansan, Vincent Ezin, Ifagbémi Bienvenue Chabi, Mathieu Anatole Tele Ayenan, Hubert Adoukonou‐Sagbadja, Aliou Saïdou, Adam Ahanchede

**Affiliations:** ^1^ Department of Crop Production, Faculty of Agricultural Sciences University of Abomey‐Calavi Cotonou Benin; ^2^ Laboratory of Human Nutrition and Valorization of Food Bio‐Ingredients, Faculty of Agricultural Sciences University of Abomey‐Calavi Cotonou Benin; ^3^ World Vegetable Center West and Central Africa Coastal and Humid Regions, IITA‐Benin Campus Cotonou Benin; ^4^ Laboratory of Genetic and Biotechnology, Faculty of Sciences and Technology University of Abomey‐Calavi Cotonou Benin

**Keywords:** *Allium cepa* L., drought stress, resilience, stress indices, yield

## Abstract

Onion productivity is sensitive to dry conditions. Breeding tolerant onion genotypes could improve productivity in regions vulnerable to water deficit stress. In this study, morphophysiological and yield parameters were used to evaluate the effects of drought on 14 genotypes at different growth stages. Three replications and two treatments—control and drought—were used in the split‐plot design experiment, which was carried out in a greenhouse. For 10, 25, and 25 days during growth, bulb initiation, and bulb development, respectively, drought was applied. The findings showed that onions' susceptibility to drought depends on the growth stage and stress level. Morphological and physiological parameters decreased dramatically as plant developmental stages varied and the stress duration increased. After 25 days of stress during bulb development, all parameters demonstrated a significant decrease (*p* < 0.001). Onions' proline content did, however, rise as a result of the drought. The study found that drought during bulb development considerably decreased yield by 33.85% (*p* < 0.001). Responses to drought stress varied among the various onion genotypes. Goudami, Prema, and Red_Jewel F1 were drought‐tolerant, while Red_Creole, AVON_1074, and Safari were sensitive, and Local, AVON_1317, and Dayo displayed intermediate tolerance. The tolerant genotypes may be useful for improving regions vulnerable to drought.

## 1 Introduction

Onion ( *Allium cepa* L.) is a C3 plant from the Alliaceae family (Ghodke et al. 2018) that requires 350–550 mm of water for optimum yield. According to FAOSTAT, the global production of onions was estimated at 111,273,599.9 tons, with an average yield of 22.5667 tons per hectare in 2023 (FAOSTAT 2024). Compared to production in previous years, onion yield declined by 0.21% from the 111,503,046.58 tons obtained in 2022. Generally, India contributes significantly to onion production, with 31,687,000 tons annually, followed by China with 24,542,011.2 tons. In West Africa, onion is cultivated on 42,911 ha with 955,617 tons. Benin produced 94,906 tons of onions, with an average yield of 11.316 tons per hectare in 2024 (DSA 2025). This yield is gradually lower than those in previous years: 12.686 tons in 2023, 13.140 tons in 2021 and 2022, and 16.898 tons per hectare in 2020.

Onions contribute to the economic world and also participate in the microeconomy of a rural region (Baloch et al. 2014; Hanci and Gökçe 2016). Nutritionally, onions play an important role in human health because the bulb contains carbohydrates, proteins, lipids, minerals, vitamins, quercetin, and polyphenolic compounds (Konate et al. 2018; Sagar et al. 2020). Onions have promising antimicrobial activity and are used in the treatment and prevention of sickness, such as hypercholesterolemia, diabetes, hypertension, and coronavirus 2 (Teshika et al. 2019; Sagar et al. 2020; Salem et al. 2023). Onion is one of the most important horticultural crops in Benin. Onion yield was estimated to be 13.140 t/ha, and the production was 79,372 tons in 2021 (DSA 2025). Annual onion production in Benin does not meet local demand, and the country relies on imports (Mensah et al. 2019). In Benin, onions are cultivated under diverse climatic conditions, but a substantial part of the onion production area is concentrated in the north of the country. Onion genotypes are produced in the dry seasons in most African countries. However, onions are susceptible to drought stress. Drought stress negatively impacts morphological parameters such as plant height, number of leaves, and leaf area, which result in significant yield losses of up to 65% in onions (Ghodke et al. 2018). Onion has a small, shallow root system with maximum roots in about 0.18 m; however, irrigation water that moves below 0.76 m is not accessible to the onion crop (Drinkwater and Janes 1955). Dry spells caused by climate variability have reduced global bulb production by approximately 30% (Gedam et al. 2021; Sansan et al. 2024). The level of damage to bulb yield varies depending on the cultivar and phenological stage during which drought stress occurs (Ghodke et al. 2018). Additionally, drought stress affects the physiological and biochemical parameters of onion plants. Many studies have described the reduction of physiological parameters, such as chlorophyll a, chlorophyll b, carotenoid, and total chlorophyll, relative water content, membrane, and stability index in onion plants cultivated under drought stress (Gedam et al. 2021; Gökçe et al. 2022; Chaudhry et al. 2023). Biochemical analyses indicated that drought stress decreases the synthesis of secondary metabolites such as phenols, flavonoids, tannins, and pyruvic acid (Ghodke et al. 2018; Vidya Vani et al. 2019) and increases the accumulation of proline in onion plants (Hanci and Cebeci 2014; Vidya Vani et al. 2019). To adapt to drought stress, plants must alter their morphological, physiological, and biochemical parameters. Under drought stress conditions, tolerant genotypes demonstrate enhanced values in several key parameters. These include chlorophyll a, chlorophyll b, carotenoid levels, total chlorophyll, relative water content, membrane stability index, and drought tolerance efficiency, which exceeds 90%. Additionally, they show improved membrane integrity, water use efficiency, antioxidant enzyme activity, and proline content (Caldwell et al. 2003; Hanci and Cebeci 2014; Gedam et al. 2021; Ezin et al. 2023). The high proline content in onions allows the plants to maintain their osmotic potential during drought stress and protects leaf membrane cells from damage. However, little knowledge is available regarding the physiological and biochemical responses of onions to drought stress (Chaudhry and Gokce 2020; Gedam et al. 2021). However, the majority of research focuses on the bulb formation stage of onions to impose water stress. This study evaluates the response of onions at various stages of development using a combination of agro‐physiological parameters and yield indices. The present study aimed to: (1) evaluate the effects of drought stress on morphological, physiological, and yield parameters of different onion genotypes, (2) explore the role of biochemical parameters in drought stress tolerance.

This study hypothesized that severe drought conditions significantly reduce chlorophyll content, chlorophyll fluorescence, plant growth, and yield. In response to drought stress, onion plants accumulate proline to maintain osmotic potential and protect leaf membrane cells from damage.

## 2 Materials and Methods

### 2.1 Plant Material

This study evaluated a total of 14 onion genotypes, which include 3 commercial genotypes obtained from the East–West Seed Company in Benin: Prema, Dayo, and Red_Jewel F1. Additionally, 5 line genotypes from WorldVeg in Mali were included: AVON_1317, AVON_1074, Goudami Violet_Galmi, and Synthetique 1. Furthermore, 5 commercial genotypes—Safari, ARES, Red_Creole, Idol, and Rouge_Tama—were provided by BENIN‐SEMENCES. Finally, a local cultivar, resulting from open pollination, was obtained from farmers in the Republic of Benin.

### 2.2 Experimental Design, Conditions, and Drought Stress Treatments

The experiment was conducted in a greenhouse at ambient temperature (18°C–28°C) and ambient humidity of 60% to 80% at the International Institute of Tropical Agriculture, Abomey‐Calavi, Benin, from June 2023 to November 2023. Seeds of fourteen onion genotypes were sown, and after 3 weeks, when the seedlings had three leaves, the plantlets were transplanted into plastic pots (height: 25 cm and diameter: 25 cm) of 12‐kg capacity filled with soil type silty sandy clay with an acidic pH. Three weeks after transplantation, drought stress was imposed using the method described by Chaudhry and Gokce (2020), with minor modifications. We suspended the water supply for 10 days, 40 days after transplantation, during the vegetative stage. Fifty‐seven days after transplantation, we stopped the water supply for 20 days, at the bulb initiation stage. Seventy‐seven days after transplantation, we stopped the water supply for 25 days, during bulb development. The plants were watered twice a day to maintain optimal soil moisture levels. Additionally, fertigation, agronomic practices, and plant protection measures were used to improve crop quality.

The irrigation management strategy was designed to ensure an adequate water supply for plant growth before the plants were subjected to drought stress.

We laid out the experiment in a split‐plot design with three replications. The main plots were the 14 genotypes (C), and the subplots were the two different groups for water supply (Control and Drought Stress). The control group (C) contained 42 plants, and the drought stress group (D) also contained 42 plants. Each genotype in the experiment included a total of 6 plants. Therefore, the total number of plants used during the entire experiment was 84.

Data on all agro‐morphological and physiological parameters was collected from both plots, and leaf samples were collected and stored for biochemical analysis.

### 2.3 Morphological and Physiological Parameters

Morphological parameters such as plant height, the number of leaves, leaf length, leaf width, and pseudo‐stem diameter were recorded on the 10th, 20th, and 25th days after imposing drought stress on both control and stressed plants.

Physiological parameters, including the chlorophyll index and chlorophyll a fluorescence, were measured in both treatment groups (control and drought stress) using the 3rd or 4th leaf of the plants, according to genotype, on the 10th, 20th, and 25th days following the imposition of drought stress. Measurements of physiological parameters were taken between 8 and 11 a.m. to minimize the effects of environmental midday stresses. After 25 days of drought stress, the relative water content was assessed.

#### 2.3.1 Leaf Chlorophyll Content

The chlorophyll content serves as an indicator of the health of onion leaves. Leaf chlorophyll index was measured using a SPAD 502 Chlorophyll‐Meter (Minolta SPAD‐502, Soil Plant Analysis Development, Minolta Co., Osaka, Japan) on both control and drought‐stressed plants. Onion leaf (3rd or 4th fresh leaf) was selected from each pot, and measurements were taken as the average of three replications.

#### 2.3.2 Chlorophyll a Fluorescence

Fluorescence data was collected from the leaves using a modulated chlorophyll fluorometer (OS1‐FL, Model OS‐30P; Opti‐Sciences, New Hampshire, USA). The data included dark fluorescence (*F*_o_), maximal fluorescence (*F*_m_), variable fluorescence (*F*_v_), and photochemical yield (*F*_v_/*F*_m_, where *F*_v_ = *F*_m_−*F*_o_). Before taking measurements, the 3rd or 4th, or fresh onion leaves were selected from each pot and dark‐adapted using cuvette clips for 45 min.

#### 2.3.3 Relative Water Content

The relative water content (RWC) was evaluated on the 3rd or 4th leaf. For each replicate, onion leaf segments of about 7 cm long were sampled, and the fresh weights (FW) were measured. Then, the samples were immersed in distilled water for 24 h and re‐weighed to determine the turgid weight (TW). Turgid leaf samples were then dried in a hot air oven at 80°C for 24 h to measure the dry weights (DW). The RWC was determined based on the procedure of Barrs and Weatherley (1962) by the formula:

$$\mathrm{RWC}\;\left(\%\right)=\frac{\mathrm{FW}–\mathrm{DW}}{\mathrm{TW}–\mathrm{DW}}\times 100$$

### 2.4 Biochemical Measurements

Biochemical parameters, including proline and total phenol content, were measured at the end of the third drought stress. Leaf samples were finely ground into powder and stored at −80°C for analysis of proline and total phenol content.

#### 2.4.1 Proline

Proline content was measured following a method of Bates et al. (1973) with minor modifications. 0.25 g of fresh leaf sample was homogenized in 10 mL of 3% aqueous sulfosalicylic acid. After 3 h, the mixture was centrifuged at 1500*g* for 10 min. We added 2 mL of glacial acetic acid and 2 mL of hydrofluoric acid to 2 mL of the supernatant and boiled the solution at 100°C in a water bath for 1 h. The solution was cooled down by placing it on ice. 4 mL of toluene was added and mixed vigorously using the vortex for 15–20 s, and the toluene containing the chromophore was separated using a separatory funnel, and the absorbance was measured at 520 nm in a spectrophotometer against an appropriate toluene blank. The proline content was determined from a standard curve prepared with L‐proline and expressed in (μmol/g FW). The unknown proline content was calculated from the samples using the standard graph. The proline concentration was then calculated using a formula:

$$\text{Proline}\;\left(\text{μmol}/g\;\mathrm{FW}\right)=\frac{e\times V1\times V2}{m\times 115.5}$$

where *e* is the μg/mL proline, V1 is the toluene volume, V2 is the volume of sulfosalicylic acid, and *m* is the weight (g) of the fresh sample used.

#### 2.4.2 Total Phenolic Content (TPC)

The total phenolic content of the leaf sample was determined using the Folin–Ciocalteu (FC) reagent according to the method of Dahmoune et al. (2014) with minor modifications. The fresh leaf sample (0.1 g) was homogenized in 10 mL of 80% aqueous methanol, and the homogenate was centrifuged at 5000 rpm for 10 min. 1 mL of the extract was mixed with 1 mL of Folin–Ciocalteu reagent. After 5 min of incubation in the dark at room temperature, 1 mL of 0.2 mM Na_2_CO_3_ solution was added, and then incubated for the next 45 min. The absorbance of the mixture was measured at 760 nm using a UV–VIS Spectrophotometer (Hitachi U‐1800, Japan). Gallic acid at different concentrations (0–200 μL mL^−1^) was used to measure the standard curve. Phenol content was expressed as mg gallic acid equivalent g^−1^ fresh weight (mg GAE g^−1^ FW). Total Phenolic Content (TPC) was determined using the formula:

$$\mathrm{TPC}\;\left(\mathrm{mg}\;\mathrm{GAE}\;ge^{-1}\;\mathrm{FW}\right)=\frac{C\times V}{m}$$

where *c* is the sample concentration from the calibration curve (mg/mL), *V* is the volume (mL) of the solvent used for the extraction, and m is the weight (g) of the fresh sample used.

### 2.5 Bulb Yield Measurements and Drought Tolerance Indices

Upon reaching maturity, the onion bulbs were harvested, and their yield parameters were evaluated. Measurements were taken for bulb diameter (mm), bulb length (mm) using digital calipers, and bulb weight (grams) using a precision weighing balance. Additionally, the indices for drought tolerance were determined (Table 1).

**TABLE 1:** Drought tolerance indices used for bulb yield.

Indices	Formula	Description
DTE (%)	$\mathrm{DTE}=\frac{\text{Yiel}d_{\text{drought}}}{\text{Yiel}d_{\text{control}}\;}\times 100$	Drought tolerance efficiency (Cabello et al. 2013)
STI	$\mathrm{STI}=\frac{\left(\text{Yiel}d_{\text{drought}}\times \text{Yiel}d_{\text{control}}\right)}{{\left(\text{Mean yield of}\;\mathrm{all}\;\text{genotype}s_{\text{control}}\right)}^{2}}$	Stress tolerance index (Fernandez 1992)
DSI	$\mathrm{DSI}=\frac{1-\frac{\text{Yiel}d_{\text{drought}}}{\text{Yiel}d_{\text{control}}}}{\text{Stress intensity}}$	Drought susceptibility index (Fischer and Maurer 1978)
SI	$\mathrm{SI}=1-\frac{\text{Mean yield of}\;\mathrm{all}\;\text{genotype}s_{\text{drought}}}{\text{Mean yield of}\;\mathrm{all}\;\text{genotype}s_{\text{control}}}$	Stress intensity (Fischer and Maurer 1978)
MRP	$\mathrm{MRP}=\frac{\text{Yiel}d_{\text{drought}}}{\text{Mean yield of}\;\mathrm{all}\;\text{genotype}s_{\text{drought}}}+\frac{\text{Yiel}d_{\text{control}}}{\text{Mean yield of}\;\mathrm{all}\;\text{genotype}s_{\text{control}}}$	Mean relative performance (Raman et al. 2012)
MP	$\mathrm{MP}=\frac{\text{Yiel}d_{\text{drought}}+\text{Yiel}d_{\text{control}}}{2}$	Mean productivity (Raman et al. 2012)

### 2.6 Statistical Analysis

To evaluate the effects of the treatments (Control and Drought) on all studied parameters, we applied Linear Mixed‐Effects Models (LMM) using the lme4 library for all parameters except leaf number, for which we used the Poisson generalized linear mixed model (GLMM). Additionally, we conducted a two‐way analysis of variance (ANOVA) to assess the impact of genotypes and drought, along with their interaction, on the parameters.

We assessed the assumptions of the ANOVA using the Shapiro–Wilk test for normality of residuals and the Levene test for homogeneity of variance. When these assumptions were not met for the variables leaf width, pseudo‐stem diameter, and chlorophyll content (SPAD), we applied logarithmic transformations. To identify significant differences in various parameters recorded in the onion genotypes under control and drought stress conditions, we used Student's *t*‐test.

Boxplots were created to visually demonstrate the impact of drought stress on the onion parameters. We analyzed correlations by calculating a two‐tailed Pearson correlation coefficient with a significance level of 0.05. Furthermore, we used Principal Component Analysis (PCA) and biplot PCA to identify the variables that most effectively discriminate between the genotypes. Cluster analysis was performed to project the variables and genotypes onto axes and to group the genotypes based on their performance under drought stress. All analyses were conducted using R software version 4.4.0 (R Core Team 2024).

## 3 Results

### 3.1 Morphological Parameters Responses in Onions to Drought Stress During Different Growth Phases

At 10 days of drought stress during the vegetative stage, the morphological parameters, including the number of leaves, leaf diameter, and pseudo‐stem diameter, decreased significantly in response to drought stress; however, the other morphological parameters, like plant height and length of leaves, recorded non‐significant differences from the control (Table S1). Under drought stress conditions, three genotypes, namely Idol, Local, and AVON_1074, recorded the highest values for the number of leaves, 5.33, 5.00, and 4.67, respectively. However, the highest values of leaf diameter were observed in genotypes Local (5.60 cm), Goudami (4.03 cm), and ARES (3.93 cm). These same genotypes had the highest values for pseudo‐stem diameter.

For 20 days of drought during bulb initiation, all morphological parameters except the length of leaves showed a significant reduction in the stressed onion plants, when compared with their controls (Table S2). The genotypes Red_Jewel F1, Prema, Dayo, and Local presented the maximum values of plant height under drought stress. Red Jewel F1, Goudami, Prema, and Local gave the best performance among genotypes subjected to drought stress for the number of leaves at bulb initiation. The highest values of pseudo‐stem diameter were obtained in genotypes Goudami, Local, Red Jewel F1, and ARES. Among the genotypes subjected to drought, the highest pseudo‐stem diameter was found in Goudami, Local, Red_Jewel F1, and ARES. Four genotypes, Dayo, Local, Prema, and Goudami, have recorded the highest leaf diameter. Plant height and length of leaves values of Red_Jewel F1 were not affected by the drought at bulb initiation because it obtained optimal values for these attributes under control and drought conditions. In onion genotypes, Dayo and Local, a significant decrease was not observed for leaf diameter. In Goudami, the pseudo‐stem diameter was not impacted by 20 days of stress.

On the 25 days of drought stress, at the bulb development stage, all morphological parameters of onion genotypes under drought stress were found to be significantly lower (*p* < 0.001) compared to the control (Table 2). Under drought conditions, Prema, Goudami, and Dayo had the highest plant height and leaf length. The genotypes Local (7.33) and Idol (6.67) recorded the highest number of leaves, followed by Dayo, ARES, and Goudami, which had the same number of leaves equal to 6.33 under drought at bulb development. The highest mean values for leaf diameter were found in Goudami (6.17 cm), Local (6.11 cm), Prema (5.53 cm), and Synthetique (5.31 cm). The genotypes Goudami (10.76 mm) and Local (10.75 mm) also outperformed the other genotypes with pseudo‐stem diameter after 25 days of stress, followed by Dayo (8.92 mm) and Red_Jewel F1 (8.75 mm).

**TABLE 2:** Mean values of morphological parameters of onion genotypes under 25 days of drought stress at the bulb development stage.

Genotypes	PH (cm)		NL		LL (cm)		LD (cm)		PD (mm)	
	Control	Drought	Control	Drought	Control	Drought	Control	Drought	Control	Drought
	54.13	45.99	7.55	5.88	48.29	41.32	6.41	4.77	9.93	7.99
*p*	0.000***		0.000***		0.000***		0.000***		0.000***	
ARES	56.43 b	45.20 cd	8.00 b	6.33 b	49.90 bcde	40.07 c	5.72 d	4.33 ab	10.60 bc	8.13 bc
AVON_1074	52.47 c	41.80 de	7.33 bcd	5.67 bc	46.83 cdef	38.77 c	5.83 d	4.83 ab	9.38 cde	7.33 bcd
AVON_1317	50.08 cd	47.23 bc	8.33 ab	6.00 bc	44.63 def	41.33 c	5.83 d	5.21 ab	10.29 bc	7.72 bc
Dayo	58.80 b	52.50 b	7.67 bc	6.33 b	51.93 bcd	47.07 b	6.55 c	4.98 ab	11.17 b	8.92 b
Goudami	67.30 a	52.80 b	9.00 a	6.33 b	59.90 a	49.13 ab	8.97 b	6.17 a	13.75 a	10.76 a
IDOL	52.50 c	47.70 bc	8.00 b	6.67 ab	46.67 cdef	42.07 c	6.04 d	4.59 ab	8.95 cde	7.59 bc
Local	67.07 a	52.47 b	9.00 a	7.33 a	60.27 a	47.07 b	8.66 b	6.11 a	13.19 a	10.75 a
Prema	58.50 b	57.17 a	7.33 bcd	6.00 bc	53.17 bc	51.90 a	9.72 a	5.53 ab	10.09 bcd	8.04 bc
Red_Creole	45.30 de	38.50 ef	7.33 bcd	5.67 bc	39.87 fg	34.70 d	4.84 e	4.17 ab	8.48 def	6.69 cd
Red_Jewel F1	60.20 b	50.03 bc	7.67 bc	6.00 bc	55.47 ab	45.47 b	6.14 d	4.95 ab	9.40 cde	8.75 bc
Rouge_Tama	41.67 e	33.33 g	5.67 e	4.00 e	37.07 g	28.40 f	4.65 ef	3.40 ab	7.06 f	5.77 d
Safari	48.83 cd	41.53 de	6.67 cd	5.00 cd	42.73 efg	38.53 c	5.69 d	4.53 ab	10.20 bc	8.50 bc
Synthetique	52.67 c	47.63 bc	7.33 bcd	6.33 b	47.20 cdef	42.27 c	6.77 c	5.31 ab	8.46 def	7.18 bcd
Violet_Galmi	46.03 de	35.93 fg	6.33 de	4.67 de	40.40 fg	31.67 e	4.34 f	2.67 b	7.99 ef	5.66 d
*p*	< 2.2e‐16***	< 2.2e‐16***	0.03837*	0.009**	< 2.2e‐16***	< 2.2e‐16***	3.807e‐05***	0.009493**	< 2.2e‐16***	< 2.2e‐16***

Abbreviations: LD, leaf diameter; LL, leaf length; NL, number of leaves; PD, pseudostem diameter; PH, plant height.

* Significatif at 5%.

** Significatif at 1%.

*** Significatif at 0.1%.

Significance: For each column the means that do not share the same alphabetic letter are significantly different (*p* < 0.05).

### 3.2 Physiological Parameters Responses in Onions to Drought Stress During Different Growth Phases

Physiological parameters such as chlorophyll content (SPAD), photochemical yield (*F*_v_/*F*_m_), and potential activity of PS II (*F*_v_/*F*_o_) were evaluated during different onion growth and development stages (Tables S3 and S4; Table 3). The onion plants under drought stress for 10 days during the growth stage showed a reduction (*p* ≤ 0.05) in chlorophyll content and photochemical yield; however, the potential activity of PSII was not significantly different from the control. The genotypes AVON1317 (65.20), Goudami (50.97), and Prema (50.93) recorded the highest values of chlorophyll content when stressed, while the genotypes Dayo (0.770), Red Jewel F1 (0.765), and Prema (0.765) showed superior performance based on the photochemical yield.

**TABLE 3:** Effect of drought stress on physiological parameters of onion genotypes under 25 days of stress.

Genotypes	SPAD		*F*_v_/*F*_m_		*F*_v_/*F*_o_	
	Control	Drought	Control	Drought	Control	Drought
	59.02	53.02	0.882	0.850	6.19	5.45
*p*	0.000***		0.000***		0.000***	
ARES	56.90 h	52.17 g	0.878 abcd	0.855 abc	6.64 ab	5.76 a
AVON_1074	54.97 i	50.93 i	0.865 bcd	0.825 cd	6.16 ab	4.70 ab
AVON_1317	60.77 c	57.87 c	0.903 abc	0.884 ab	6.88 ab	6.71 a
Dayo	60.67 c	59.10 a	0.914 ab	0.888 ab	6.55 ab	6.65 a
Goudami	60.97 b	55.63 e	0.925 a	0.903 a	6.12 ab	6.01 a
IDOL	59.60 e	49.57 l	0.890 abc	0.8477 abc	5.17 b	4.87 ab
Local	74.30 a	58.90 b	0.905 abc	0.890 a	6.12 ab	5.17 ab
Prema	60.03 d	56.03 d	0.911 ab	0.895 a	7.59 a	6.23 a
Red_Creole	57.60 g	49.07 m	0.866 bcd	0.860 abc	6.44 ab	5.25 ab
Red_Jewel F1	54.13 k	51.63 h	0.861 bcd	0.851 abc	6.20 ab	5.84 a
Rouge_Tama	59.03 f	50.00 k	0.836 d	0.740 e	5.41 b	3.45 b
Safari	56.57 i	50.67 j	0.895 abc	0.832 bcd	5.50 b	5.44 ab
Synthetique	60.77 c	53.50 f	0.855 cd	0.847 abc	5.88 ab	5.54 ab
Violet_Galmi	50.07 l	47.20 n	0.854 cd	0.790 d	5.99 ab	4.64 ab
*p*	0.004671**	0.04263*	7.893e‐11***	< 2.2e‐16***	0.002742**	2.016e‐05***

*Note:* *, ** and ***indicate differences at *p* ≤ 0.05, *p* ≤ 0.01, and *p* ≤ 0.001 probability level.

Abbreviations: *F*_v_/*F*_m_, photochemical yield; *F*_v_/*F*_o_, potential activity of PS II; SPAD, chlorophyll content.

Significance: For each column the means that do not share the same alphabetic letter are significantly different (*p* < 0.05).

Drought stress of 20 days imposed at the stage of bulb initiation significantly decreased the chlorophyll content and photochemical yield of genotypes. The maximum chlorophyll content was observed in genotypes Synthetique (60.03), Safari (59.93), and AVON_1317 (59.90); however, Goudami (0.913), Red_Creole (0.880), and Prema (0.877) showed the highest photochemical yield among all other genotypes at bulb initiation.

Under 25 days of drought stress at the bulb development stage, all physiological parameters were significantly reduced (*p* < 0.001) compared to the control. We noted after 25 days of drought imposition that Dayo, Local, and AVON_1317 had the highest chlorophyll content. The genotypes Goudami (0.903), Prema (0.895), and Local (0.860) recorded the highest photochemical yield. We observed that the genotype ‘Goudami’ outperformed the other genotypes with the least decrease in photochemical yield after 25 days of drought stress. AVON_1317, Dayo, and Prema had the highest PSII.

### 3.3 Biochemical Parameters Responses in Onions to Drought Stress at Bulb Development Phases

**FIGURE 1:**
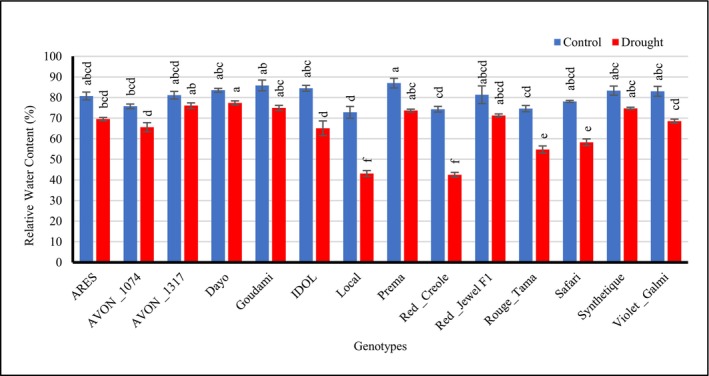
Effect of drought stress on the relative water contents of different onion genotypes. Data followed by different letters are significantly different at *p* ≤ 0.05.

Relative water contents of onion genotypes significantly decreased (*p* < 0.001) in all onion genotypes subjected to 25 days of drought during the bulb development compared to their performance under well‐watered conditions. Under drought stress, the genotypes Dayo (77.36%), AVON_1317 (76.05%), Goudami (74.96%), Synthetique (74.72%), Prema (73.64%), and Red_Jewel F1 (71.31%) had the highest values for the relative water contents (RWC) (Figure 1).

**FIGURE 2:**
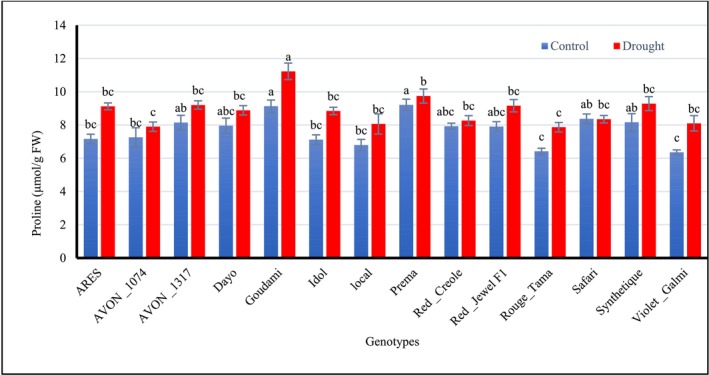
Effect of drought stress on the proline contents of different onion genotypes. Data followed by different letters are significantly different at *p* ≤ 0.05.

However, drought stress imposed for 25 days during bulb development significantly increased (*p* < 0.001) proline contents of onion genotypes. Goudami (11.23 μmol/g FW) and Prema (9.74 μmol/g FW) had accumulated the highest proline contents, followed by genotypes Synthetique (9.29 μmol/g FW) and AVON_1317 (9.20 μmol/g FW) under drought stress during the bulb development stage (Figure 2).

**FIGURE 3:**
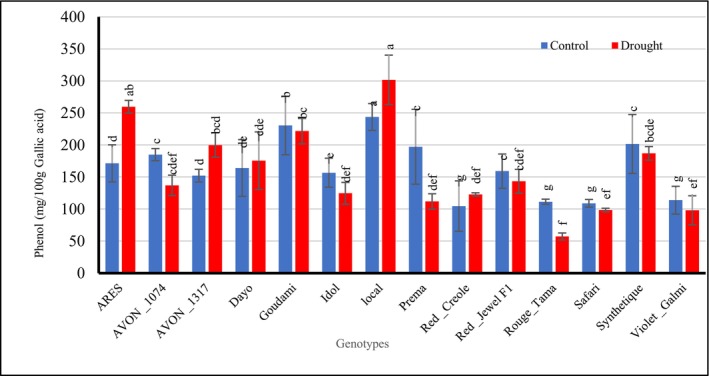
Effect of drought stress on the total phenolic contents of different onion genotypes. Data followed by different letters are significantly different at *p* ≤ 0.05.

There was no significant variation (*p* ≥ 0.05) in total phenolic contents between onion plants stressed and non‐stressed onion plants, but genotypes Local, ARES, and Goudami presented the maximum values, respectively, after 25 days of drought during bulb development (Figure 3).

### 3.4 Bulb Yield and Drought Tolerance Indices Parameters Associated With Onion Genotypes

Our study showed that the drought stress during the bulb development significantly reduced onion bulb parameters, particularly the bulb yield (*p* < 0.001) (Table 4). For example, the average bulb yield was significantly decreased by 33.85% compared to the control treatment. Additionally, drought stress reduced bulb calibers and size. Prema (45.07 mm), Red_Jewel F1 (44.67 mm), and Goudami (41.07 mm) had the largest bulb diameter, while Red_Jewel F1, Dayo, and IDOL had the biggest bulb size (Table 4).

**TABLE 4:** Effect of drought stress on the yield parameters of onion genotypes.

Genotypes	BD (mm)		BL (mm)		BW (g)	
	Control	Drought	Control	Drought	Control	Drought
	40.49	35.42	50.98	44.99	57.50	38.86
*p*	0.000***		0.002**		3.16e‐10***	
ARES	42.70 cd	34.93 bcd	56.57 abc	47.27 ab	66.37 b	36.40 c
AVON_1074	41.33 cde	33.17 bcd	42.57 c	33.80 b	48.43 de	27.67 d
AVON_1317	42.20 cde	35.60 bcd	48.47 abc	43.07 ab	62.73 bc	41.93 b
Dayo	36.93 efg	30.97 cd	59.47 ab	52.80 ab	51.60 d	38.13 bc
Goudami	45.59 bc	41.07 ab	50.99 abc	44.90 ab	73.23 a	60.66 a
IDOL	43.03 cd	38.10 bc	64.30 a	50.70 ab	53.93 d	37.60 bc
Local	46.27 bc	33.80 bcd	49.50 abc	45.77 ab	53.37 d	40.27 bc
Prema	51.93 a	45.07 a	52.03 abc	47.03 ab	70.83 a	57.17 a
Red_Creole	33.63 g	22.97 e	52.90 abc	36.80 ab	39.80 f	22.23 e
Red_Jewel F1	48.33 ab	44.67 a	61.43 ab	54.70 a	76.13 a	60.23 a
Rouge_Tama	33.63 g	29.47 d	46.33 bc	37.37 ab	44.60 e	19.53 e
Safari	38.20 defg	30.87 cd	47.10 bc	38.53 ab	51.77 d	30.33 d
Synthetique	39.93 def	35.37 bcd	50.83 abc	49.27 ab	59.50 c	41.50 b
Violet_ Galmi	35.53 fg	27.47 de	41.90 c	37.17 ab	52.73 d	30.33 d
*p*	2.65e‐10***	2.491e‐08***	0.000***	0.009**	4.368e‐16***	< 2.2e‐16***

Abbreviations: BD, bulb diameter; BL, bulb length; BW, weight bulbs; ns, no significant.

* Significatif at 5%.

** Significatif at 1%.

*** Significatif at 0.1%.

Significance: For each column the means that do not share the same alphabetic letter are significantly different (*p* < 0.05).

Based on drought indices, genotypes were classified into four groups according to percent bulb yield reduction and drought tolerance efficiency (Table 5). Three onion genotypes, Goudami (17.17%), Prema (19.29%), and Red_Jewel F1 (20.89%), recorded the minimum yield reduction percentage values and the highest values for drought tolerance efficiency, and they were categorized as highly tolerant to drought. Five others genotype such as Local (24.55%), Dayo (26.10%), Synthetique, IDOL and AVON_1317 had yield reduction percentages between 24% and 35% and drought tolerance efficiencies from 70% to 60% and were classified as tolerant genotypes, whereas, 5 genotypes, namely Safari, Violet_Galmi, AVON1074, Red_Creole and ARES recorded 40% yield reduction with drought tolerance efficiency of 50% and were identified as moderately tolerant genotypes. The onion Rouge_Tama recorded a 56.21% yield reduction with 43.79 DTE under drought, and it was characterized as a sensitive genotype.

**TABLE 5:** Drought tolerance indices of onion genotypes.

Genotypes	DTE (%)	YLD (%)	STI	DSI	MRP	MP
ARES	54.84	45.16	0.73	1.41	2.09	51.39
AVON_1074	57.13	42.87	0.41	1.34	1.55	38.05
AVON_1317	66.84	33.16	0.80	1.04	2.17	52.33
Dayo	73.90	26.10	0.60	0.82	1.88	44.87
Goudami	82.83	17.17	1.34	0.54	2.83	66.95
IDOL	69.72	30.28	0.61	0.95	1.91	45.77
Local	75.45	24.55	0.65	0.77	1.96	46.82
Prema	80.71	19.29	1.22	0.60	2.70	64.00
Red_Creole	55.85	44.15	0.27	1.38	1.26	31.02
Red_Jewel F1	79.11	20.89	1.39	0.65	2.87	68.18
Rouge_Tama	43.79	56.21	0.26	1.76	1.28	32.07
Safari	58.59	41.41	0.47	1.29	1.68	41.05
Synthetique	69.75	30.25	0.75	0.95	2.10	50.50
Violet_Galmi	57.52	42.48	0.48	1.33	1.70	41.53
Mean	66.15	33.85	0.71	1.06	2.00	48.18

Abbreviations: DSI, drought susceptibility index; DTE, drought tolerance efficiency; MP, mean productivity; MRP, mean relative performance; STI, stress tolerance index; YLR, yield reduction.

### 3.5 Effect of Genotypes and Drought Stress on Morphological, Physiological, and Yield Parameters

The analysis of variance (ANOVA) indicated that water supply and genotypes were statistically significant (*p* < 0.001) for all morpho‐physiological parameters jointly on the 10th, 20th, and 25th days of drought stress, except for the potential activity of PSII (*F*_v_/*F*_o_) for 20 days (Table 6). The interaction between genotypes and water regime was only significant for photochemical yield (*F*_v_/*F*_m_) (*p* < 0.001) under 10 days of stress. Chlorophyll content, photochemical yield, and potential activity of PSII were significantly affected by the interactions between treatments and genotypes during 20 days of drought stress, while plant height, leaf length, and photochemical yield were significantly reduced under 25 days of drought stress.

**TABLE 6:** Analysis of variance for morphological and physiological parameters of onions under drought stress.

Days_Stress	Source	df	PH	NL	LL	LD	PD	SPAD	*F*_v_/*F*_m_	*F*_v_/*F*_o_
10 days	Genotypes (G)	13	486.77***	66.23***	298.26***	866.51***	504.62***	436.67***	605.991**	87.86***
	Treatments (T)	1	16.23***	23.00***	11.01***	33.07***	39.76***	38.59***	48.752***	8.95**
	G*T	13	1.76^ns^	3.42^ns^	2.74^ns^	16.54^ns^	11.70^ns^	11.19^ns^	64.925***	13.65^ns^
20 days	Genotypes (G)	13	259.63***	346.23***	170.90***	110.76***	566.15***	46.20***	52.971***	186.40***
	Treatments (T)	1	26.16***	86.95***	8.83**	18.46***	38.10***	90.16***	10.789**	0.18^ns^
	G*T	13	16.88^ns^	14.49^ns^	15.09^ns^	4.34^ns^	5.70^ns^	58.59***	33.068**	51.01***
25 days	Genotypes (G)	13	818.73***	46.59***	600.38***	56.8***	422.65***	66.74***	225.132***	64.54***
	Treatments (T)	1	291.10***	46.21***	172.83***	26.44***	153.13***	34.91***	52.808***	20.30***
	G*T	13	64.94***	2.64^ns^	37.83***	8.28^ns^	15.23^ns^	12.64^ns^	32.801**	14.22^ns^

Abbreviations: *F*_v_/*F*_m_, photochemical yield; *F*_v_/*F*_o_, potential activity of PS II; LD, leaf diameter; LL, leaf length; NL, number of leaves; ns, no significant; PD, pseudostem diameter; PH, plant height; SPAD, chlorophyll content.

* Significatif at 5%.

** Significatif at 1%.

*** Significatif at 0.1%.

The analysis of variance (ANOVA) for biochemical and yield parameters of onion genotypes indicated that the main effects of the two independent factors were found to be significant (*p* < 0.05) for all parameters in the study, except for phenolic compound content and proline, for which water regime and genotypes had no significant effect, respectively (Table 7). The interactions of the two factors were significant for relative water content, bulb diameter, and weight.

**TABLE 7:** Analysis of variance of biochemical and yield parameters of onion genotypes.

Source	df	RWC	Proline	Phenol	BD	BL	BW
Genotypes (G)	13	21.44***	0.86^ns^	98.95***	290.98***	78.26***	513.87***
Treatments (T)	1	507.43***	10.89**	0.18^ns^	60.93***	18.64***	47.57***
G*T	13	2.64**	14.77^ns^	19.30^ns^	53.67***	14.77^ns^	32.91**

Abbreviations: BD, bulb diameter; BL, bulb length; BW, bulb weight; ns, no significant; RWC, relative water content.

* Significatif at 5%.

** Significatif at 1%.

*** Significatif at 0.1%.

**FIGURE 4:**
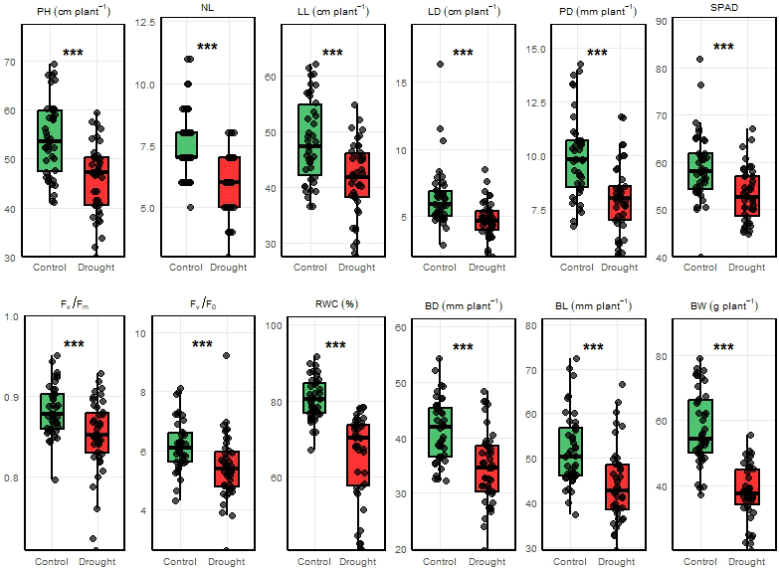
The descriptive statistics of morphological, physiological, and yield parameters of onion genotypes under drought stress at the bulb development stage are displayed in the box plots. Different asterisk(s) on the boxes indicate a significant difference between growth conditions. *** indicates significant at *p* ≤ 0.001, according to the LSD test. BD, bulb diameter; BL, bulb length; BW, bulb weight; SPAD, chlorophyll content; LD, leaf diameter; LL, leaf length; *F*_v_/*F*_m_, maximum quantum yield; NL, number of leaves; PH, plant height; *F*_v_/*F*_o_, Potential activity of PS II; PD, pseudostem diameter; RWC, relative water content.

A box plot was used to visualize the descriptive statistics of the morphological, physiological, and yield parameters at the bulb development stage (Figure 4). Due to drought stress, all morphological, physiological, and yield parameters decreased significantly (*p* ≤ 0.001) over the control conditions. Drought stress decreased plant height (PH), number of leaves (NL), leaf length (LL), leaf diameter (LD), and pseudostem diameter (PD) by 15.0%, 22.1%, 14.4%, 25.6%, and 19.6%, respectively, over control, whereas 10.2%, 3.65%, 12.0%, 18.7%, 16.5%, 14.5%, and 34.1% decreases were recorded in chlorophyll content (SPAD), maximum quantum yield (*F*_v_/*F*_m_), potential activity of PS II (*F*_v_/*F*_o_), relative water content (RWC), bulb diameter (BD), bulb length (BL), and bulb weight (BW), respectively (Figure 4).

**FIGURE 5:**
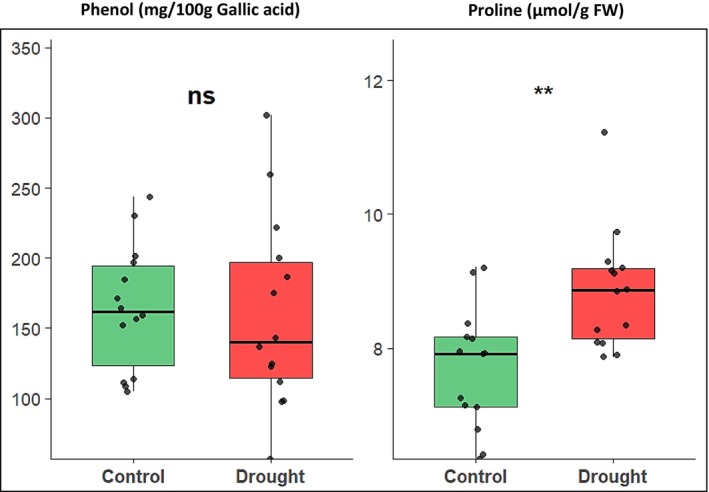
Boxplots of biochemical parameters of onion genotypes under drought stress at the bulb development stage. Different asterisk(s) on the boxes indicate a significant difference between growth conditions. ** indicates significant at *p* ≤ 0.01, according to the LSD test. ns, no significant.

Concerning biochemical parameters, there was no significant difference in phenol content between control and drought stress conditions; however, drought stress significantly increased the proline compared to control (Figure 5).

### 3.6 Relationships of Parameters Subjected to Drought Conditions

**FIGURE 6:**
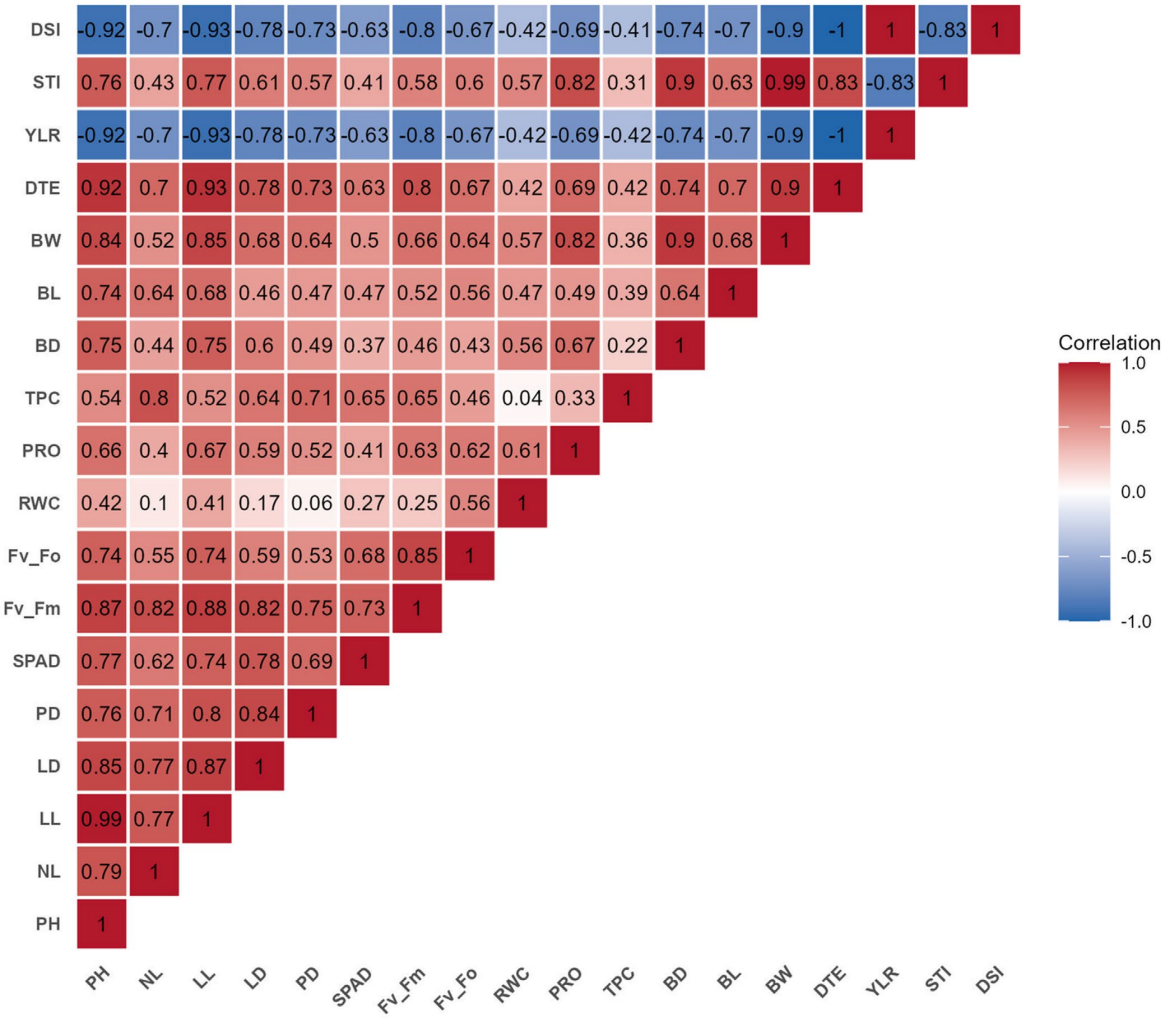
Pearson correlation coefficients among the morpho‐physiological traits under drought stress at the bulb development stage. BD, bulb diameter; BL, bulb length; BW, bulb weight; SPAD, chlorophyll content; DSI, drought susceptibility index; DTE, drought tolerance efficiency; LD, leaf diameter; LL, leaf length; *F*_v_/*F*_m_, maximum quantum yield; NL, number of leaves; PH, plant height; *F*_v_/*F*_o_, potential activity of PS II; Pro, proline; PD, pseudostem diameter; RWC, relative water content; STI, stress tolerance index; TPC, total phenol content; YLR, yield reduction.

Plant height and leaf length (0.99***), leaf length and leaf diameter (0.87***), plant height and leaf diameter (0.85***), leaf diameter and pseudostem diameter (0.84***), leaf length and pseudostem diameter (0.80***), plant height and number of leaves (0.79***), number of leaves and leaf length (0.77***), number of leaves leaf diameter (0.77***), plant height and pseudostem diameter (0.76***), number of leaves and pseudostem diameter (0.71***) were significantly and positively correlated (Figure 6). Plant height was positively associated with drought tolerance efficiency (0.92***), maximum quantum yield (0.87***), and bulb weight (0.84***), but showed a negative correlation with yield reduction percentage (−0.92***) and drought susceptibility index (−0.92***) (Figure 6). The number of leaves was positively associated with maximum quantum yield (0.82***) and proline (0.80***). The leaf diameter showed a positive relation with maximum quantum yield (0.82***) and drought tolerance efficiency (0.78***). Bulb diameter had a strong and positive relationship with bulb weight (0.90***) and stress tolerance index (0.90***), but had a negative correlation with yield reduction (−0.74***) and drought susceptibility index (−0.74***). Bulb length was negatively correlated with yield reduction (−0.70***) and drought susceptibility index (−0.70***). Bulb weight had a significant and positive relationship with stress tolerance index (0.99***) and drought tolerance efficiency (0.90***), but showed a strong negative correlation with yield reduction percentage (−0.90***) and drought susceptibility index (−0.90***). The drought tolerance efficiency exhibited a positive relationship with stress tolerance index (0.83***), but exhibited a strong negative correlation with yield reduction percentage (−1.00***) and drought susceptibility index (−1.00***). The yield reduction percentage had a strong association with drought susceptibility index (1.00***), but showed a negative correlation with stress tolerance index (−0.83***). The stress tolerance index exhibited a negative relationship with drought susceptibility index (−0.83***). The proline had a positive association with bulb weight (0.82***), stress tolerance index (0.82***), drought tolerance efficiency (0.69***), and bulb diameter (0.64**), but had a negative association with yield reduction percentage (−0.69***) and drought susceptibility index (−0.69***). The relative water content was positively linked to proline (0.61**) (Figure 6). The maximum quantum yield had a strong and positive association with potential activity of PS II (0.85***), drought tolerance efficiency (0.80***), but showed a negative correlation with yield reduction percentage (−0.80***) and drought susceptibility index (−0.80***). The chlorophyll content was positively linked to maximum quantum yield (0.73***), and potential activity of PS II (0.68***) (Figure 6).

### 3.7 Principal Component Analysis and Ascending Hierarchical Classification

Under drought stress during the growth stage, the graph of variables showed that the first two components cumulatively explained 71.91% of variability (Figure S1a). Different parameters contributed both positively and negatively to different PC groups. The parameters such as PD (0.638), LL (0.625), LD (0.595), and PH (0.592) positively contributed to the PC1, while *F*_v_/*F*_o_ (0.563) and *F*_v_/*F*_m_ (0.352) were positively associated with the second component. The projection of the morphophysiological and genotypic variables showed that the genotypes Local, Dayo, and Goudami, Red_Jewel F1, and Prema had positive PC1 values, indicating the best performance in terms of pseudostem diameter, leaf diameter, leaf length, and plant height. The genotypes AVON_1317 and IDOL contributed to the formation of the second component, resulting in increased *F*_v_/*F*_m_ and *F*_v_/*F*_o_ ratios (Figure S1b).

Under drought stress at the bulb initiation stage, the first two components explained 70.4% of the total variation (Figure S2a). The plant height (PH), number of leaves (NL), leaf length (LL), pseudostem diameter (PD), and the leaf diameter (LD) contributed very positively to PC1, while SPAD, *F*_v_/*F*_m_, and *F*_v_/*F*_o_ were positively associated with PC2 (Figure S2a). The PCA biplot revealed that Red_Jewel F1, Prema, Goudami, and Dayo had the highest PH, NL, LL, PD, and LD. The genotypes Synthetique and AVON1317 were characterized by high SPAD and *F*_v_/*F*_m_ (Figure S2b).

**FIGURE 7:**
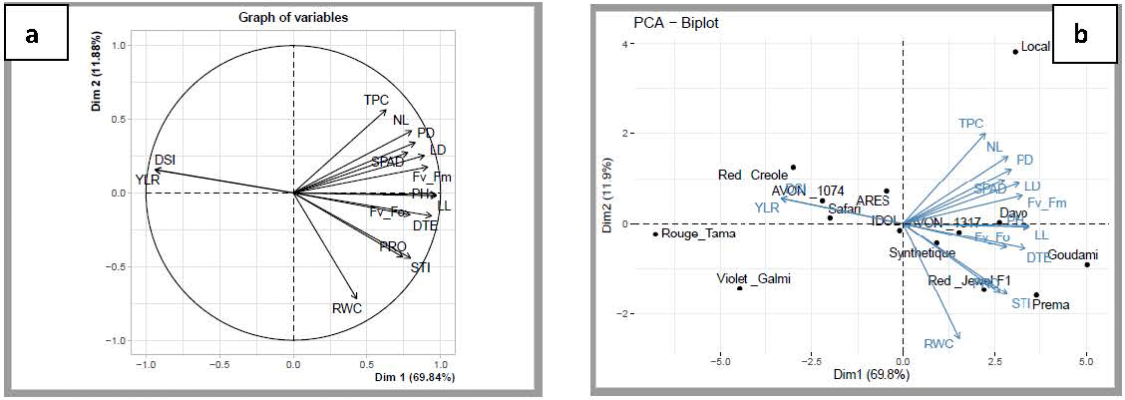
PCA of variables (a) and Principal component biplot presenting grouping of 14 onion genotypes and distribution of morpho‐physiological traits under drought stress at the bulb development stage (b).

Under drought stress during the bulb development stage, the first two components explained 81.72% of the total variability (Figure 7a). The leaf length, plant height, drought tolerance efficiency, leaf diameter, photochemical yield, potential activity of PSII, and chlorophyll content contributed to the PC1; however, the drought susceptibility index and yield reduction negatively contributed to the same component. The total phenolic content, number of leaves, and pseudo‐stem diameter were positively correlated with PC2, but the relative water content, stress tolerance index, and proline content were negatively associated with this principal component. The biplot of the principal component analysis revealed that Goudami, Prema, Red Jewel F1, Dayo, and AVON1317 were characterized by high LL, PD, LD, PH, SPAD, *F*_v_/*F*_o_, RWC, proline, DTE, and STI. The genotype Local had the highest values of NL and TPC. The genotypes Red Creole, AVON1074, and Safari were characterized by a low tolerance to drought stress as exemplified by the association with high yield reduction (YLR) and drought susceptibility index (DSI) (Figure 7b).

Cluster analysis grouped the 14 genotypes into three clusters using Ward's method of clustering, based on the square Euclidean distance between different parameters.

Hierarchical tree of genotypes under drought stress at the vegetative growth showed that cluster III includes Local, Dayo, and Goudami, which recorded significantly higher mean values for plant height (43.23), leaf diameter (4.30), pseudostem diameter (5.46) leaf length (39.99) than other genotypes in clusters II and I (Figure S3).

Compared to the other two clusters, cluster III, composed of Local, Red Jewel F1, Prema, Goudami, and Dayo, showed the highest mean values for all morphological parameters, followed by AVON_1317, IDOL, Synthetique, and ARES of cluster II during bulb initiation (Figure S4).

**FIGURE 8:**
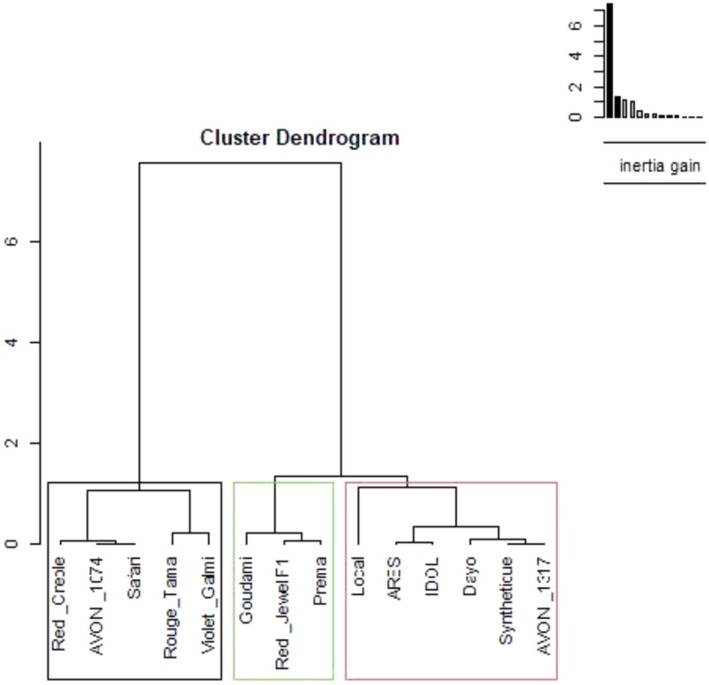
Hierarchical tree under drought stress during the bulb development.

Under drought stress at the bulb development, the genotypes Goudami, Prema, and Red Jewel F1 grouped in cluster III and were characterized by high values for the stress tolerance index (1.14), drought tolerance efficiency (79.14), leaf length (48.39), plant height (53.12), proline content (9.75) and *F*_v_/*F*_o_ (6.18). Dayo, Local, IDOL, ARES, Synthetique, and AVON_1317 of cluster II recorded higher mean values for TPE and the number of leaves. The percentage of yield reduction (45.42%) and the index of sensitivity to drought (1.42) were very high in the genotypes Rouge Tama, Violet Galmi, Red Creole, AVON_1074, and Safari of cluster I (Figure 8).

## 4 Discussion

Our findings revealed that the susceptibility of onions to drought stress varied depending on the growth stage and the duration of drought stress. We found that drought stress during 10 days significantly reduced the number of leaves, leaf diameter, and pseudo‐stem diameter of all genotypes at the vegetative stage. However, during bulb initiation, all morphological parameters except the length of leaves significantly decreased by 20 days of drought stress, and at the bulb development stage, all morphological parameters of onion genotypes under 25 days of drought stress were found to be considerably lower compared to the control. The reduction observed during the vegetative stage was less significant than for the other stages, indicating that onions are less sensitive to drought stress during the vegetative stage than during the bulb initiation and establishment stages. Furthermore, the plants exhibited a cumulative response to drought stress throughout their development. This indicates that plants exposed to drought during bulb initiation were already stressed at the vegetative stage, and the same was observed during bulb establishment. These results correlate with the previous studies that reported that onion is more sensitive to drought stress during bulb formation and development than during the vegetative stage (Khokhar 2017).

After 25 days of drought stress, the genotypes Goudami, Local, and Prema had the highest values for morphological parameters. Furthermore, the prolonged duration of drought stress and the transition across growth phases imposed increasingly severe constraints, ultimately restricting overall onion plant growth. Similar results were reported in onion field and greenhouse experiments where 20–45 days of drought negatively affected morphological parameters such as leaf number, length, width, and area, with a high rate of leaf senescence (Ghodke et al. 2018; Chaudhry and Gokce 2020; Gökçe et al. 2022). Reduced plant growth under drought may be caused by reduced cell division, cell differentiation, and expansion thus leading to a reduction in growth, notably in the number of leaves, plant height, leaf width, and length (Hayat et al. 2008; Farooq et al. 2009; Sousaraei et al. 2021), which constitutes the morphological adaptation mechanism to drought.

Drought stress also negatively affected physiological parameters. The results showed that when the duration of drought increases, the reduction in physiological parameters increases gradually. Two physiological parameters, notably the chlorophyll content and the photochemical yield (*F*_v_/*F*_m_), were slightly reduced by a drought duration of 10 days at the growth stage, but the chlorophyll content decreased significantly at the bulb initiation when the drought duration was 20 days. All physiological parameters, including chlorophyll content (SPAD), photochemical yield (*F*_v_/*F*_m_), and potential PSII activity (*F*_v_/*F*_o_), decreased by 25 days of drought stress during the bulb development phase. These reductions may be because onion plants use most of their energy to synthesize osmolytes, namely proline and phenolics, which enable them to resist drought. Decreased chlorophyll content varies depending on the duration of drought stress imposed. A decrease in chlorophyll content under drought stress was also reported in previous studies on onion (Hanci and Cebeci 2014; Chaudhry and Gokce 2020; Gökçe et al. 2022), strawberry cultivars (Ödemiş et al. 2020), and rice (Phunthong and Pitaloka 2024). During the drought stress, the decrease in chlorophyll content might be due to the degradation of chlorophyll biosynthetic enzymes. This reduction in assimilative pigment levels during the growth phase causes a decrease in photosynthetic activity and stomatal conductance, resulting in low organic matter synthesis in plants (Arief et al. 2023; Bhusal et al. 2020).

The photochemical quantum yield of photosystem II (*F*_v_/*F*_m_) is a very important variable that is measured when all centers are open (Zhang et al. 2019). It is used to identify PSII damage and possible photoinhibition in the leaves of plants under drought (Rahbarian et al. 2011; Ezin et al. 2021, 2023). In this study, drought stress induced a reduction of *F*_v_/*F*_m_ and *F*_v_/*F*_o_, aligning with previous findings on onion (Semida et al. 2020) and tomato (Sousaraei et al. 2021; Ahmad et al. 2023). The decrease in *F*_v_/*F*_m_ was an explanation for the reduction in Rubisco activity and the partial inactivation of PSII during drought stress (Zhang et al. 2019). The recent development of chlorophyll a fluorescence (ChlF) may be a potentially valuable new approach to determining the photochemical efficiency of leaves, specifically, providing detailed information on the status and function of photosystem II (PSII) reaction centers, light‐harvesting antenna complexes, and both the donor and acceptor sides of PSII (Kalaji et al. 2016; Song et al. 2024). Recently, some investigators used chlorophyll fluorescence parameters to evaluate the fluorescence response to drought in Acer genotypes (Banks 2018), in cucumbers (Song et al. 2024), and found that *F*_v_/*F*_m_ was significantly decreased under drought stress. In our study, *F*_v_/*F*_m_ was able to help unravel the tolerance status of the genotypes to drought stress.

Relative water content is an important physiological parameter to estimate plant water status under drought stress. Here, the relative water contents (RWC) of onion genotypes considerably decreased in all onion genotypes exposed to 25 days of drought during bulb development compared to their respective controls. For instance, the relative water content decreased by 18.7% under 25 days of drought stress during the bulb development stage. Drought stress negatively impacted the leaf water status of plants (Ghodke et al. 2018; Hassan et al. 2023). The reduction in leaf water content could result in decreased metabolic reactions and photosynthetic activity. However, genotypes responded differently to drought stress, and genotypes Dayo, AVON_1317, Goudami, Synthetique, Prema, and Red Jewel F1 maintained higher leaf water content, which is an indication of the ability to absorb water from the soil and conserve water in the leaf during periods of drought stress. Under drought stress conditions, high relative water content (RWC) in onion plants allows high values for leaf area, total chlorophyll content, antioxidant enzyme activity, membrane stability index, and bulb yield (Gedam et al. 2021).

In response to a decreasing water status in the leaf, plants accumulate more osmolytes, like proline. Thus, proline content was significantly higher under drought stress of 25 days at bulb development as reported in previous studies (Gökçe et al. 2022, 2023; Hanci and Cebeci 2014). The highest proline content was observed in genotypes Goudami, Prema, Synthetique, and AVON_1317, under drought stress. Maximum proline was recorded in genotype Prema under drought stress in India (Vidya Vani et al. 2019). This increase in proline helps the plant adapt to drought stress. Several recent studies have shown that proline accumulation plays a crucial role in stressed plants, including osmotic pressure regulation and maintenance of protein and cell membrane stability (Chun et al. 2018; Laura et al. 2020; Wang et al. 2022).

Moreover, drought stress causes oxidative stress in the plant. Thus, the synthesis of phenolic secondary metabolites increased in response to the oxidative damage caused by drought. Our results showed that total phenolic content was significantly higher in genotypes such as Local, ARES, and Goudami and lower in others under drought conditions compared to watered plants. The increase in phenol in certain genotypes corroborates the findings of Vidya Vani et al. (2019) and Ghodke et al. (2018). The plant accumulates higher total phenolics in response to drought stress as one of the tolerance mechanisms.

Drought stress significantly decreased onion bulb yield and yield components (Zheng et al. 2012; Wakchaure et al. 2018; Nurga et al. 2020; Gedam et al. 2021). We found that the drought stress during bulb development significantly reduced onion bulb parameters, particularly the bulb yield (*p* < 0.001), with a reduction of 33.85% compared to the control treatments. In comparison to our findings, a lower (14%) and a higher (65%) yield reduction due to drought stress was reported by Almaroai and Eissa (2020) and Ghodke et al. (2018), respectively. These differences in the magnitude of yield reduction across studies could be due to differences in the time of stress imposition, duration of stress, and growth stage at which plants were exposed to drought. Yield reduction could stem from a decrease in photosynthetic activity of the leaves and low translocation of photosynthate products to bulb growth. Drought‐tolerant genotypes maintained their water status, produced more proline, and regulated their photosynthesis with high photochemical yield (*F*_v_/*F*_m_) and high potential PSII activity (*F*_v_/*F*_o_).

We grouped onion genotypes into three clusters based on their parameters and drought tolerance indices. Genotypes Goudami, Prema, and Red Jewel F1 were grouped in cluster III and are tolerant to drought stress. Dayo, Local, IDOL, ARES, Synthetique, and AVON1317 from cluster II were considered moderate drought‐tolerant genotypes. They have higher mean values for TPE and the number of leaves. Red_Tama, Violet_Galmi, Red_Creole, AVON1074, and Safari from cluster I have high yield reduction and drought sensitivity and are drought sensitive. Tolerant onion genotypes under drought stress are characterized by high proline accumulation (Chaudhry et al. 2023) and high RWC, DTE, and STI (Gedam et al. 2021). In contrast, sensitive onion genotypes under drought stress accumulate low levels of proline and lower water content (Ghodke et al. 2020). Overall, the results of this study are consistent with our findings regarding onions under drought stress during bulb development, which showed that water stress negatively impacts morphological, physiological, and yield parameters (Sansan et al. 2025).

These findings highlight the importance of understanding the sensitive phase of onion development in relation to drought stress. The practical application of the results could be in terms of irrigation timing recommendations in regions where water is scarce.

## 5 Conclusion

All onion genotypes exhibited varying responses to drought stress. Drought stress negatively impacted all morphological and physiological parameters, as well as bulb yield, while simultaneously increasing proline content. The identification of drought tolerance and sensitivity was facilitated by analyzing morphophysiological and biochemical parameters, along with stress indices.

Biplot analysis revealed that the drought‐tolerant genotypes—Goudami, Prema, and Red_Jewel F1—demonstrated high values for leaf length (LL), pseudostem diameter (PD), leaf diameter (LD), plant height (PH), SPAD readings, *F*_v_/*F*_o_ ratios, relative water content (RWC), proline levels, drought tolerance efficiency (DTE), and stress tolerance indices (STI). These genotypes performed well under drought conditions, showing the best agronomic, physiological, and biochemical parameters, as well as tolerance indices. Therefore, Goudami, Prema, and Red_Jewel F1 are recommended for onion growers and could be valuable for future improvement programs in drought‐prone areas. In contrast, the Red_Creole genotype exhibited significantly lower yields and was more severely affected by drought conditions. As such, Prema, Goudami, and Red_Jewel F1 are the preferred varieties for onion cultivation in these challenging climates.

## 6 Future Directions

The main challenge in understanding the drought tolerance mechanism of onion plants is identifying the drought tolerance mechanisms of genotypes under different conditions. Therefore, future research on onion drought tolerance should focus on identifying genes responsible for drought resistance, understanding the underlying mechanisms, and developing drought‐tolerant varieties.

## Funding

The authors have nothing to report.

## Conflicts of Interest

The authors declare no conflicts of interest.

## Supporting information


**Figure S1:** PCA of variables (a) and Principal component biplot presenting grouping of 14 onion genotypes and distribution of morpho‐physiological traits under drought stress at vegetative growth (b).
**Figure S2:** PCA of variables (a) and Principal component biplot presenting grouping of 14 onion genotypes and distribution of morpho‐physiological under drought stress at the bulb initiation (b).
**Figure S3:** Hierarchical tree under drought stress during vegetative growth.
**Figure S4:** Hierarchical tree under drought stress during bulb initiation.

**Table S1:** Mean values of parameters for onion genotypes under 10 days of drought stress at the vegetative growth.
**Table S2:** Mean values of characters for onion genotypes under 20 days of drought stress during stage bulb initiation.
**Table S3:** Effect of drought stress on physiological parameters of onion genotypes under 10 days stress.
**Table S4:** Effect of drought stress on physiological parameters of onion genotypes under 20 days stress.

## Data Availability

The data that supports the findings of this study is available in the Supporting Information of this article.
