# Supplementary material for: Response of Elite Onion Genotypes to Drought Stress: Morphophysiological and Agronomic Parameters and Stress Indexes
Source: Plant Environ Interact. 2025 Nov 28;6(6):e70099. doi: 10.1002/pei3.70099 (PMC12661220; doi:10.1002/pei3.70099)
Supplement: Supplementary file 1 — Figure S1: PCA of variables (a) and Principal component biplot presenting grouping of 14 onion genotypes and distribution of morpho‐physiological traits under drought stress at vegetative growth (b). Figure S2: PCA of variables (a) and Principal component biplot presenting grouping of 14 onion genotypes and distribution of morpho‐physiological under drought stress at the bulb initiation (b). Figure S3: Hierarchical tree under drought stress during vegetative growth. Figure S4: Hierarchical tree under drought stress during bulb initiation. [file PEI3-6-e70099-s002.docx]

**Supplementary Figures**


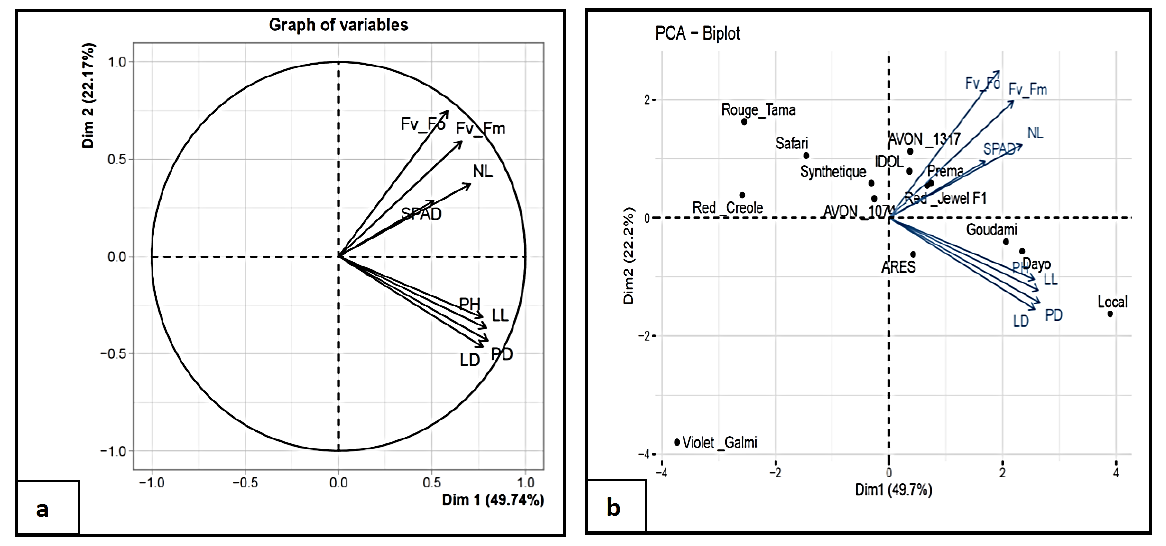


**Supplementary Figure 1:** PCA of variables (a) and Principal component biplot presenting grouping of 14 onion genotypes and distribution of morpho-physiological traits under drought stress at vegetative growth (b)

**
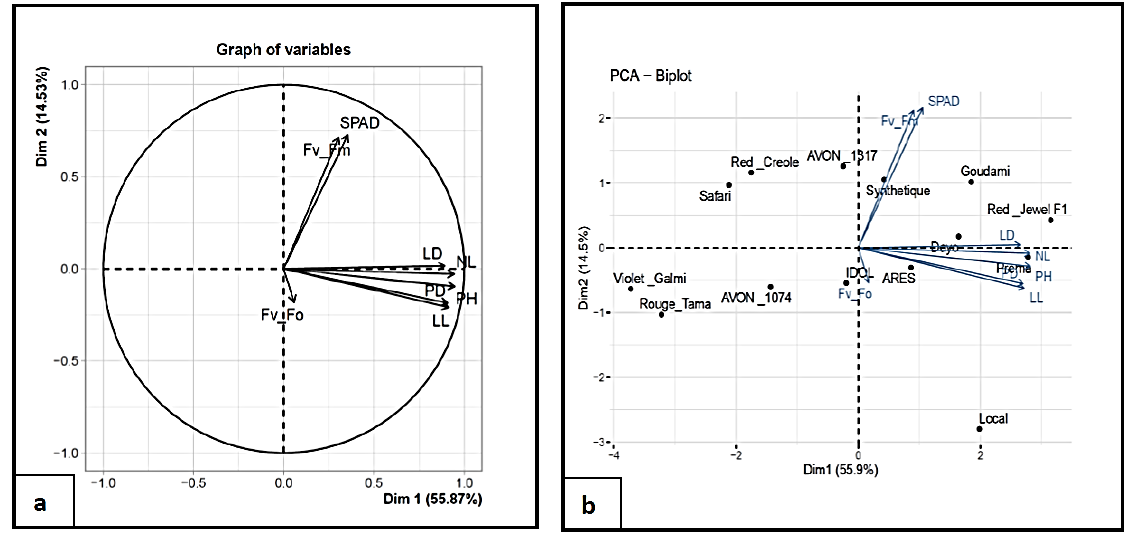
**

**Supplementary Figure 2:** PCA of variables (a) and Principal component biplot presenting grouping of 14 onion genotypes and distribution of morpho-physiological under drought stress at the bulb initiation (b).

**
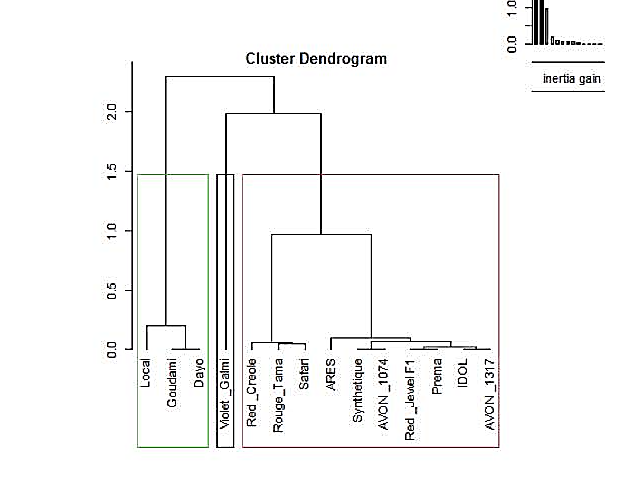
**

**Supplementary Figure 3:** Hierarchical tree under drought stress during vegetative growth.

**
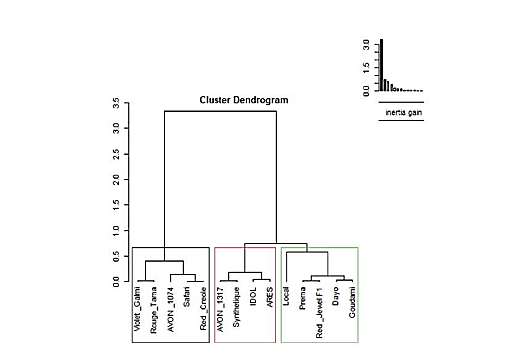
**

**Supplementary Figure 4:** Hierarchical tree under drought stress during bulb initiation.
