# Supplementary material for: Response of Elite Onion Genotypes to Drought Stress: Morphophysiological and Agronomic Parameters and Stress Indexes
Source: Plant Environ Interact. 2025 Nov 28;6(6):e70099. doi: 10.1002/pei3.70099 (PMC12661220; doi:10.1002/pei3.70099)
Supplement: Supplementary file 2 — Table S1: Mean values of parameters for onion genotypes under 10 days of drought stress at the vegetative growth. Table S2: Mean values of characters for onion genotypes under 20 days of drought stress during stage bulb initiation. Table S3: Effect of drought stress on physiological parameters of onion genotypes under 10 days stress. Table S4: Effect of drought stress on physiological parameters of onion genotypes under 20 days stress. [file PEI3-6-e70099-s001.docx]

**Supplementary Tables**

**Supplementary Table 1. Mean values of parameters for onion genotypes under 10 days of drought stress** **at** **the vegetative growth**

| **Genotypes** | **PH (cm)** | | **NL** | | **LL (cm)** | | **LD (cm)** | | **PD (mm)** | |
| --- | --- | --- | --- | --- | --- | --- | --- | --- | --- | --- |
|  | **Control** | **Drought** | **Control** | **Drought** | **Control** | **Drought** | **Control** | **Drought** | **Control** | **Drought** |
|  | 37.47 | 35.47 | 4.86 | 4.17 | 34.39 | 32.18 | 3.47 | 3.16 | 4.42 | 3.81 |
| **p_value** | 0.122 ^ns^ | | 0.000 *** | | 0.118 ^ns^ | | 0.010** | | 0.003** | |
| **ARES** | 34.13 ef | 32.07 efg | 4.67 b | 4.33 bcd | 35.27 bcd | 33.63 bc | 4.03 c | 3.93 b | 5.37 b | 4.80 b |
| **AVON _1074** | 37.87 cdef | 36.20 cde | 5.33 ab | 4.67 abc | 31.96 bcde | 29.93 cd | 3.40 d | 2.93 cd | 4.12 cde | 3.47 bcde |
| **AVON _1317** | 36.43 def | 34.53 defg | 5.33 ab | 4.33 bcd | 30.77 cde | 30.13 cd | 3.13 d | 2.73 cd | 4.47 bcd | 3.70 bcde |
| **Dayo** | 48.13 a | 46.37 a | 4.67 b | 4.00 cd | 46.40 a | 44.57 a | 3.47 d | 3.27 c | 5.20 b | 4.47 bc |
| **Goudami** | 44.70 ab | 42.77 b | 5.33 ab | 4.33 bcd | 37.63 bcd | 35.77 bc | 4.60 b | 4.03 b | 5.10 b | 4.73 b |
| **IDOL** | 37.03 def | 35.13 def | 5.67 a | 5.33 a | 30.57 cde | 29.43 cd | 3.37 d | 2.93 cd | 4.67 bc | 4.03 bcd |
| **Local** | 43.47 abc | 40.57 bc | 5.33 ab | 5.00 ab | 40.53 b | 39.63 ab | 5.43 a | 5.60 a | 7.03 a | 7.20 a |
| **Prema** | 37.10 def | 34.60 defg | 5.33 ab | 4.33 bcd | 39.60 bc | 37.83 b | 3.53 d | 3.33 c | 3.70 def | 3.10 def |
| **Red _Creole** | 32.60 f | 29.93 g | 4.67 b | 3.67 d | 29.07 de | 25.47 d | 2.53 e | 2.07 e | 3.47 ef | 2.70 ef |
| **Red _Jewel F1** | 39.50 bcde | 37.23 cd | 5.00 ab | 4.00 cd | 39.13 bc | 35.80 bc | 3.27 d | 2.87 cd | 4.10 cde | 3.83 bcd |
| **Rouge_Tama** | 25.07 g | 24.83 h | 4.33 b | 3.67 d | 23.87 e | 19.57 e | 2.37 e | 2.40 de | 3.07 f | 2.70 ef |
| **Safari** | 31.83 f | 30.66 fg | 4.33 b | 4.00 cd | 31.60 bcde | 29.50 cd | 3.10 d | 2.47 d | 3.70 def | 2.37 f |
| **Synthetique** | 40.90 bcd | 38.33 cd | 4.67 b | 4.00 cd | 33.07 bcd | 29.03 cd | 3.20 d | 2.77 cd | 3.70 def | 2.97 def |
| **Violet _Galmi** | 35.80 def | 33.43 defg | 3.33 c | 2.67 e | 32.07 bcde | 30.17 cd | 3.20 d | 2.93 cd | 4.17 cde | 3.30 cde |
| **p_value** | < 2.2e-16 *** | < 2.2e-16 *** | 0.023 * | 0.002** | < 2.2e-16 *** | < 2.2e-16 *** | < 2.2e-16 *** | < 2.2e-16 *** | < 2.2e-16 *** | < 2.2e-16 *** |

Plant Height (PH). Number of Leaves (NL). Leaf Length (LL). Leaf Diameter (LD). Pseudostem Diameter (PD). * significatif at 5%; ** Significatif at 1%; *** significatif at 0,1%, ns: no significant.

**Supplementary Table 2. Mean values of characters for onion genotypes under 20 days of drought stress** **during stage bulb initiation**

|  | **PH (cm)** | | **NL** | | **LL (cm)** | | **LD (cm)** | | **PD (mm)** | |
| --- | --- | --- | --- | --- | --- | --- | --- | --- | --- | --- |
| **Genotypes** | **Control** | **Drought** | **Control** | **Drought** | **Control** | **Drought** | **Control** | **Drought** | **Control** | **Drought** |
|  | 60.19 | 55.10 | 10.95 | 8.95 | 52.25 | 49.06 | 7.11 | 6.08 | 16.45 | 14.57 |
| p_value | 0.01299** | | 0.000*** | | 0.08751 ^ns^ | | 0.00877 ** | | 0.02885* | |
| ARES | 66.63 ab | 59.50 bc | 11.33 d | 9.33 de | 62.17 ab | 53.37 bc | 7.13 ab | 6.30 def | 18.50 bcde | 17.10 abc |
| AVON _1074 | 56.50 bcde | 48.33 cdef | 10.33 e | 8.33 f | 51.37 bcd | 41.37 ef | 7.00 ab | 5.87 ef | 14.53 fgh | 12.43 d |
| AVON _1317 | 59.77 bcd | 55.17 bcde | 10.67 e | 8.67 ef | 50.00 bcd | 46.47 cde | 6.53 ab | 6.37 de | 15.20 efgh | 11.67 de |
| Dayo | 64.13 ab | 62.43 ab | 10.67 e | 9.33 de | 56.27 abcd | 52.97 bc | 9.33 a | 7.80 a | 17.33 cdef | 16.37 bc |
| Goudami | 63.23 ab | 55.83 bcde | 13.67 a | 11.33 b | 53.13 abcd | 49.40 cd | 9.10 a | 6.87 bc | 22.67 a | 20.57 a |
| IDOL | 54.93 bcde | 55.87 bcde | 12.00 c | 9.67 d | 47.37 cd | 46.10 cde | 7.30 ab | 5.80 f | 16.77 defg | 13.93 cd |
| Local | 64.97 ab | 61.80 ab | 13.67 a | 10.67 c | 53.73 abcd | 59.20 ab | 8.57 a | 7.57 a | 21.23 ab | 20.17 a |
| Prema | 73.77 a | 66.70 ab | 11.33 d | 10.67 c | 59.23 abc | 64.03 a | 7.90 ab | 7.13 b | 20.43 abc | 18.20 ab |
| Red _Creole | 60.70 bc | 44.33 ef | 8.33 f | 6.33 g | 44.53 d | 42.20 def | 6.53 ab | 5.77 f | 13.67 gh | 11.37 de |
| Red _Jewel F1 | 72.83 a | 71.70 a | 13.00 b | 12.33 a | 65.20 a | 63.27 a | 7.47 ab | 6.67 cd | 19.33 bcd | 17.33 abc |
| Rouge_Tama | 51.43 cde | 46.67 def | 8.00 f | 6.00 g | 48.70 bcd | 44.00 def | 4.43 b | 3.57 i | 12.23 h | 10.63 de |
| Safari | 48.93 de | 44.53 ef | 8.33 f | 6.67 g | 45.40 cd | 41.00 ef | 6.13 ab | 5.13 g | 12.83 h | 11.63 de |
| Synthetique | 57.97 bcd | 56.87 bcd | 14.00 a | 10.00 cd | 50.97 bcd | 47.33 cde | 7.60 ab | 6.167 def | 16.43 defg | 14.30 cd |
| Violet _Galmi | 46.90 e | 41.73 f | 8.00 f | 6.00 g | 43.50 d | 36.13 f | 4.50 b | 4.10 h | 9.20 i | 8.33 e |
| p_value | 2e-07 *** | 8.29e-08 *** | 3.386e-10 *** | 2.644e-07 *** | 0.00019 *** | 3.33e-07 *** | 0.004 ** | 0.000 *** | 3.02e-11 *** | 1.01e-10 *** |

Plant Height (PH). Number of Leaves (NL). Leaf Length (LL). Leaf Diameter (LD). Pseudostem Diameter (PD). * significatif at 5%; ** Significatif at 1%; *** significatif at 0,1%, ns: no significant.

**Supplementary Table 3. Effect of drought stress on physiological parameters of onion genotypes under 10 days stress**

| **Genotypes** | **SPAD** | | **Fv/Fm** | | **Fv/Fo** | |
| --- | --- | --- | --- | --- | --- | --- |
|  | **Control** | **Drought** | **Control** | **Drought** | **Control** | **Drought** |
|  | 49.51 | 44.88 | 0.762 | 0.749 | 3.12 | 2.94 |
| p_value | 0.0142 * | | 0.022 * | | 0.051 ^ns^ | |
| ARES | 49.30 cd | 45.70 b | 0.765 abc | 0.727 e | 3.68 | 3.10 |
| AVON _1074 | 46.47 cdef | 39.07 cd | 0.774 abc | 0.740 cde | 3.31 | 3.05 |
| AVON _1317 | 67.00 a | 65.20 a | 0.768 abc | 0.747 bcd | 3.29 | 3.06 |
| Dayo | 53.30 bcd | 48.00 b | 0.786 ab | 0.770 a | 3.28 | 3.06 |
| Goudami | 54.53 bc | 50.97 b | 0.786 ab | 0.763 ab | 3.23 | 3.02 |
| IDOL | 39.80 ef | 37.13 d | 0.762 bc | 0.754 abcd | 3.22 | 3.04 |
| Local | 47.60 cde | 45.2 b | 0.761 bc | 0.760 abc | 3.13 | 3.07 |
| Prema | 54.37 bc | 50.93 b | 0.788 a | 0.765 ab | 3.13 | 2.99 |
| Red _Creole | 37.70 f | 35.93 d | 0.750 c | 0.736 de | 3.11 | 2.85 |
| Red _Jewel F1 | 51.10 bcd | 48.4 b | 0.766 abc | 0.765 ab | 3.06 | 3.10 |
| Rouge_Tama | 49.33 cd | 43.37 bc | 0.763 bc | 0.746 bcd | 3.06 | 3.11 |
| Safari | 44.17 def | 35.33 d | 0.764 bc | 0.764 ab | 2.91 | 2.96 |
| Synthetique | 58.87 b | 49.13 b | 0.764 bc | 0.757 abc | 2.89 | 2.92 |
| Violet _Galmi | 39.67 ef | 33.93 d | 0.671 d | 0.693 f | 2.36 | 1.82 |
| p_value | 9.49e-09 *** | 7.64e-12 *** | 6.355e-13 *** | 1.954e-10 *** | 0.3793 ^ns^ | 0.4452 ^ns^ |

Chlorophyll content (SPAD). Photochemical yield (Fv/Fm). Potential activity of PS II (Fv/Fo). *, **, and *** indicate differences at p ≤ 0.05, p ≤ 0.01, and p ≤ 0.001 probability level. ns indicates not significant difference.

**Supplementary Table 4. Effect of drought stress on physiological parameters of onion genotypes under 20 days stress**

| **Genotypes** | **SPAD** | | **Fv/Fm** | | **Fv/Fo** | |
| --- | --- | --- | --- | --- | --- | --- |
|  | **Control** | **Drought** | **Control** | **Drought** | **Control** | **Drought** |
|  | 68.51 | 56.75 | 0.870 | 0.849 | 7.25 | 7.19 |
| p_value | 0.000*** | | 0.016 * | | 0.821 ^ns^ | |
| ARES | 77.50 c | 56.97 | 0.866 | 0.837 abc | 8.45 a | 6.40 cd |
| AVON _1074 | 85.27 b | 52.70 | 0.873 | 0.853 abc | 7.99 ab | 7.65 abc |
| AVON _1317 | 65.20 f | 59.90 | 0.864 | 0.858 abc | 6.21 de | 6.15 cd |
| Dayo | 63.80 g | 56.97 | 0.891 | 0.877 ab | 8.14 a | 7.92 ab |
| Goudami | 62.40 i | 57.37 | 0.871 | 0.913 a | 8.07 ab | 6.56 bcd |
| IDOL | 60.97 m | 54.37 | 0.856 | 0.859 abc | 7.72 abc | 8.65 a |
| Local | 85.87 a | 54.47 | 0.850 | 0.753 c | 5.20 e | 7.34 abc |
| Prema | 63.73 g | 57.73 | 0.887 | 0.877 ab | 7.66 abc | 8.41 a |
| Red _Creole | 63.40 h | 58.43 | 0.880 | 0.880 ab | 8.38 a | 8.57 a |
| Red _Jewel F1 | 70.10 e | 59.53 | 0.875 | 0.873 ab | 6.96 bcd | 6.90 bcd |
| Rouge_Tama | 61.80 k | 50.03 | 0.856 | 0.841 abc | 6.21 de | 5.35 d |
| Safari | 74.83 d | 59.93 | 0.843 | 0.825 abc | 5.47 e | 6.04 cd |
| Synthetique | 61.20 l | 60.03 | 0.874 | 0.855 abc | 6.64 cd | 6.14 cd |
| Violet _Galmi | 63.07 i | 56.03 | 0.894 | 0.791 bc | 8.40 a | 8.56 a |
| p_value | 2.361e-11 *** | 0.44 | 0.2424 | 1.171e-06 *** | 3.53e-06 *** | 2.14e-07 *** |

Chlorophyll content (SPAD). Photochemical yield (Fv/Fm). Potential activity of PS II (Fv/Fo). *, **, and *** indicate differences at p ≤ 0.05, p ≤ 0.01, and p ≤ 0.001 probability level. ns indicates not significant difference.
